# Urinary Dysfunction in Progressive Supranuclear Palsy Compared with Other Parkinsonian Disorders

**DOI:** 10.1371/journal.pone.0149278

**Published:** 2016-02-17

**Authors:** Tatsuya Yamamoto, Fuyuki Tateno, Ryuji Sakakibara, Shogo Furukawa, Masato Asahina, Tomoyuki Uchiyama, Shigeki Hirano, Yoshitaka Yamanaka, Miki Fuse, Yasuko Koga, Mitsuru Yanagisawa, Satoshi Kuwabara

**Affiliations:** 1 Department of Neurology, Chiba University Graduate School of Medicine, Chiba, Japan; 2 Department of Urology, Chiba University Graduate School of Medicine, Chiba, Japan; 3 Department of Molecular Diagnosis, Chiba University Graduate School of Medicine, Chiba, Japan; 4 Neurology Division, Department of Internal Medicine, Sakura Medical Center, Toho University, Sakura, Japan; 5 Department of Neurology, Continence Center, Dokkyo Medical University, Tochigi, Japan; Taipei Veterans General Hospital, TAIWAN

## Abstract

**Background:**

Autonomic urinary dysfunction affects patients with progressive supranuclear palsy (PSP); however, the severity and prevalence of urinary dysfunctions in these patients compared with those observed in patients with Parkinson’s disease (PD) and multiple system atrophy (MSA) are unknown.

**Objective:**

We compared urinary dysfunction characteristics in patients with PSP, PD, and MSA.

**Patients and Methods:**

Forty-seven patients who satisfied the probable or possible criteria of the National Institute for Neurological Diseases and Stroke and Society for PSP were assessed using the urinary symptoms questionnaire and the urodynamic study at Chiba and Toho Universities (*n* = 26 and 21, respectively). The results were compared with those of patients with PD and MSA (*n* = 218 and 193, respectively).

**Results:**

The mean disease duration of PSP and the mean age were 2.97 ± 0.26 and 71.4 ± 0.88 years, respectively. The mini-mental state examination and frontal assessment battery scores were 22.6 ± 0.70 and 10.7 ± 0.49, respectively. Urinary storage and voiding symptoms were observed in 57% and 56% of patients with PSP, respectively. Detrusor overactivity in the urodynamic study was detected in 81% of patients with PSP, which was slightly more than that found in patients with PD (69%) and MSA (67%); however, this was not statistically significant. Postvoid residual volume in patients with PSP was significantly more than that in patients with PD (*P* < 0.01), but was equivalent to that in patients with MSA.

**Conclusions:**

The present study demonstrated that patients with PSP experienced various urinary dysfunctions. Urinary storage dysfunction in patients with PSP was not different from that in patients with PD or MSA, whereas urinary voiding dysfunction in patients with PSP was milder than that in patients with MSA and more severe than that in patients with PD. These features should be taken into account for the differentiation of PSP from PD and MSA.

## Introduction

Progressive supranuclear palsy (PSP) is a neurodegenerative disease clinically characterized at onset by impaired balance, slowness in movement, subtle personality changes, such as apathy and disinhibition, impairment in executive functioning, bulbar symptoms, and impaired eye motion [1]. These symptoms are typically heterogeneously presented in patients with PSP. Further, the differential diagnosis of PSP, Parkinson’s disease (PD), and multiple system atrophy (MSA) presents additional challenges [2]. A subset of patients with PSP respond well to L-3,4-dihydroxyphenylalanine (L-DOPA) therapy, at least in the early stages, and may be clinically diagnosed as PD [2], whereas certain patients with MSA-Parkinsonism (MSA-P) have very mild autonomic symptoms with poor L-DOPA-responsive Parkinsonism during the early stages [3] and may be clinically diagnosed as PSP-Parkinsonism (PSP-P).

Although autonomic dysfunctions are generally milder in patients with PSP than in PD and MSA, some patients with PSP suffer from urinary symptoms [4–7]. Mild or late-onset urinary disturbances are known to occur in a percentage of patients with PSP according to the National Institute for Neurological Diseases and Stroke and Society for PSP (NINDS-SPSP) [8]; however, the exact prevalence and severity of urinary dysfunction in PSP are unknown.

Recent functional imaging and experimental animal studies suggest the existence of a number of higher micturition centers regulating micturition reflex in the cerebral cortex, basal ganglia, and brainstem [9, 10]. In particular, the prefrontal cortex regulates the voluntary control of the micturition reflex [9, 10]; as such, forebrain lesions such as tumors and vascular anomalies are known to cause urinary dysfunction [11]. The basal ganglia and brainstem are other important regulators of the micturition reflex. We have previously reported the presence of neurons that control bladder contraction and relaxation in the substantia nigra, subthalamic nucleus, and striatum in the normal cat [12–14]. The micturition reflex is controlled by spino-bulbo-spinal reflex with the periaqueductal gray (PAG) and pontine micturition centers in the brainstem playing significant roles [9, 15].

Frontal dysfunction that is detected by low frontal assessment battery (FAB) scores and reductions in cerebral blood flow in the frontal cortex is frequently observed in patients with PSP [16]. In addition, neuropathological examinations demonstrate neuronal loss and/or tau protein accumulation within the PSP frontal cortex [17]. Neuropathological changes have also been reported in the substantia nigra, striatum, medial globus pallidus, subthalamic nucleus, and several brainstem nuclei [1].

Altogether, these clinical and pathological findings suggest that dysfunction of the frontal cortex, basal ganglia, and brainstem nuclei in PSP may contribute to urinary dysfunction. Given that the urinary dysfunction in patients with PD and MSA is well recognized [18, 19], it is clinically important to understand the differences in urinary dysfunction observed in patients with PSP, MSA, and PD.

In this retrospective study, a primary goal was to concisely determine the characteristics of urinary dysfunction in patients with PSP using the urinary symptom questionnaire and the urodynamic study. We also compared our findings to the characteristics of urinary dysfunction experienced by patients with PD and MSA. We further aimed to clarify the relationship between the frontal and urinary dysfunction.

## Methods

### Standard protocol approvals, registrations, and patient consents

This study was approved by the Chiba University and Toho University Hospital institutional review committees. Approval from an ethical standards committee to conduct this study was received. Oral informed consent was obtained from all patients, and the contents of oral informed consent were recorded in medical records. We did not obtain written informed consent from all participants as this was a retrospective study, and we could not obtain written informed consent from some patients who were transferred to other hospitals or were diseased at the time of analysis. In addition, this study included only the questionnaires and examinations that are routinely performed for all parkinsonian patients. Institutional review committees in Chiba and Toho Universities approved this consent procedure for the retrospective study.

### Patient characteristics

We recruited 47 patients (39 males, 8 females) who fulfilled the probable or possible criteria of the NINDS-SPSP [8]. These patients received the urinary symptoms questionnaire and underwent the urodynamic study at Chiba and Toho Universities (*n* = 26 and 21, respectively) between January 2000 and September 2014. We evaluated urinary dysfunctions in patients with PSP by the urinary symptoms questionnaire and urodynamic study and compared the results with those of patients with PD (*n* = 218: 134 males, 84 females; mean age, 66.2 ± 0.46 years; mean duration, 3.2 years) and patients with MSA *(n =* 193: 117 males, 76 females; mean age, 64.1 ± 0.53 years; mean duration, 3.2 years) who were diagnosed at Chiba University. All patients in this study were diagnosed and evaluated by the questionnaire within a month of the urodynamic study.

### Neurological and radiological examination

All patients in this study were interviewed and examined in depth by neurologists with expertise in movement disorders. Patients with PD satisfied the United Kingdom Parkinson’s Disease Society Brain Bank clinical diagnostic criteria [20]. Patients with MSA were diagnosed as having probable or possible MSA according to Gilman’s second consensus criteria [21].

Motor functions were evaluated by the Hoehn and Yahr scale in patients with PSP. The mini-mental state examination (MMSE) and FAB were performed in all patients with PSP. Brain magnetic resonance imaging was performed in all patients with PSP and MSA. Cerebral blood flow was examined by *n*-isopropyl-*p*-[^123^I]-iodoamphetamine-single photon emission computed tomography (IMP-SPECT) in all patients with PSP diagnosed at Chiba University.

### Urinary symptoms questionnaire

We administered our original questionnaire on urinary symptoms to all patients with PSP, PD, and MSA at Chiba and Toho Universities [19, 22, 23]. After oral informed consent was obtained from all patients, the questionnaire was handed to each patient and completed by them. The questionnaire included several bladder symptoms. Each symptom, as described in the responses, was evaluated as normal, mild (>once/month), moderate (>once/week), or severe (>once/day). Our original questionnaire was used in several of our previous studies [19, 22,23].

### Urodynamic examination

Urodynamic studies were performed in all patients with an urodynamic computer (Janus; Lifetec Inc., Houston, TX, USA) in which postvoid residual (PVR) volume was measured by catheterization. Abnormal urodynamic findings included detrusor overactivity (DO), which is defined as involuntary detrusor contractions during the filling phase. Abnormal urodynamic findings during the voiding phase included impaired bladder contractility and detrusor–external sphincter dyssynergia (DSD); DSD was detected by anal sphincter electromyography (EMG). The degree of impaired bladder contractility was evaluated by Schäfer’s nomogram. The methods of the urodynamic study conformed to the standards recommended by the International Continence Society [24]. In addition, the methods of the urodynamic study were equivalent to those reported in several of our previous studies [19, 22, 23]. Identical urodynamic computers were used in Chiba and Toho Universities.

### External anal sphincter EMG

We performed a quantitative motor unit potential (MUP) analysis using an EMG computer (Neuropack Sigma MEB-5504; Nihon Kohden, Tokyo, Japan). A disposable concentric needle electrode (diameter, 0.46 mm; Alpine Biomed, Skovlunde, Denmark) was inserted into the most superficial layer of the anal sphincter muscle under audio guidance. The needle was then inserted into the right (5 o’clock position) and left (7 o’clock position) sphincter muscles, and the MUP analysis was performed separately, with five MUPs recorded on each side. The position of the needle electrode was tuned until continuous firing activities of 3–5 MUPs were visually detected. The MUPs were stored on the computer when the amplitude reached the threshold manually determined by the examiner. After visual confirmation of continuously firing 3–5 MUPs, the examiner manually sets the threshold by moving the cursor to detect 3–5 MUPs, and a total of 64 MUPs were stored on the computer. The stored MUPs were classified into the four most similar MUPs by the auto MUP analysis software. Since this auto MUP analysis software cannot identify late components (i.e., separate satellite potentials), the examiner manually set the cursor to include late components, which were included in the total MUP duration. These procedures were repeated by moving the position of the electrode to acquire 10 different MUPs. Thereafter, the mean duration, number of phases, and amplitude were calculated by the EMG computer. This external anal sphincter (EAS)-EMG method and the diagnostic criteria for neurogenic changes were identical to those from our previous studies [19, 23, 24]. The EAS-EMG method, diagnostic criteria for neurogenic changes, and the EMG computer were identical in Chiba and Toho Universities.

### Statistical analysis

The commercially available IBM SPSS Statistics Version 22.0 (IBM, Armonk, USA) software was used for statistical analysis. The unpaired *t* test was used to compare the PVR and mean duration of MUPs in patients with PSP with those in patients with PD and MSA. The unpaired *t* test was also used to compare the FAB score, MMSE, mean disease duration, and Hoehn and Yahr scale between patients with PSP with DO and those without DO. The chi-square test was used to compare the prevalence of DO and DSD in patients with PSP with that in patients with PD and MSA. The Mann–Whitney *U* test was used to compare the urinary symptoms and the degree of detrusor contraction by Schäfer’s nomogram in patients with PSP with those in patients with PD and MSA. The Mann–Whitney *U* test was also used to examine the relationship between the degree of bladder contractility and FAB score, MMSE, mean disease duration, and Hoehn and Yahr scale. Spearman’s correlation coefficient was used for the calculation of the relationship among the FAB score, MMSE, mean disease duration, Hoehn and Yahr scale, and the degree of bladder contractility. All data were expressed as means ± standard error of mean.

## Results

The mean age and mean disease duration of PSP were 71.4 ± 0.88 and 2.97 ± 0.26 years, respectively. The MMSE and FAB scores were 22.6 ± 0.70 and 10.7 ± 0.49, respectively. The mean Hoehn and Yahr scale was 3.14 ± 0.14. The decreased blood flow in the frontal lobe was observed in 22 patients with PSP (84%) diagnosed using IMP-SPECT at Chiba University. Two patients with PSP, two patients with PD, and seven patients with MSA were taking anticholinergic drugs, and 16 patients with MSA and one patient with PD were taking alpha-blockers. Eight patients with MSA also used clean intermittent self-catheterization.

### Urinary symptoms

**Fig 1:**
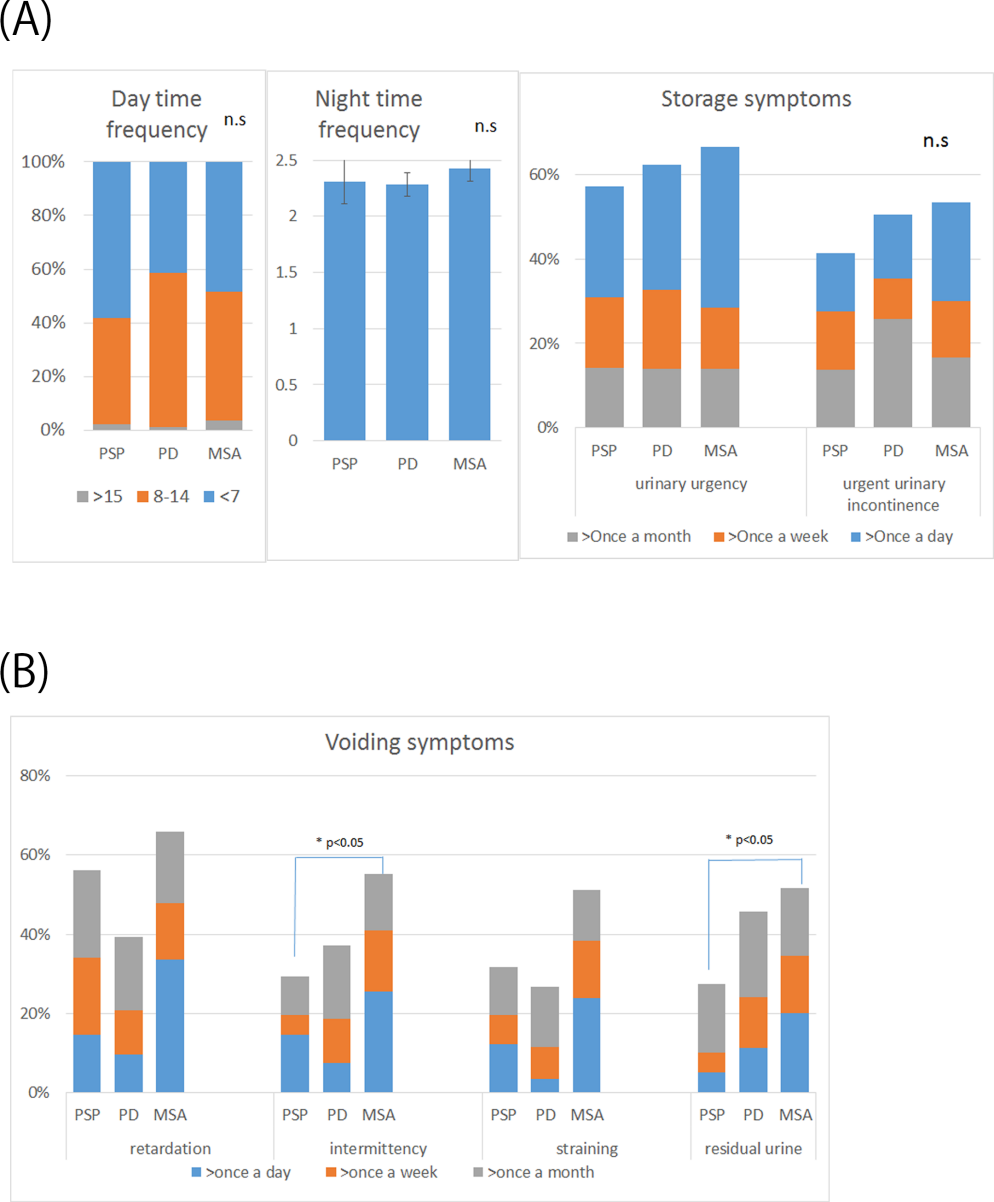
Urinary symptoms of patients with PSP, PD, and MSA assessed by the urinary symptom questionnaire. The prevalence and severity of urinary storage symptoms did not show significant differences among patients with PSP, PD, and MSA. Urinary voiding symptoms were significantly more prevalent and severe in patients with MSA than those in patients with PSP. Abbreviations: MSA, multiple system atrophy; PD, Parkinson’s disease; PSP, progressive supranuclear palsy.

Urinary storage symptoms were observed in 57% of patients with PSP, whereas urinary voiding symptoms were observed in 56% of patients with PSP. The characteristics of urinary storage symptoms in patients with PSP were similar to those observed in patients with PD and MSA (Fig 1A). In contrast, urinary voiding symptoms such as residual urine and intermittency were significantly more prevalent and more severe in patients with MSA than those in patients with PSP (*P < 0.05, Fig 1B); however, urinary voiding symptoms were equivalent between patients with PSP and PD (Fig 1B).

### Urodynamic study

**Fig 2:**
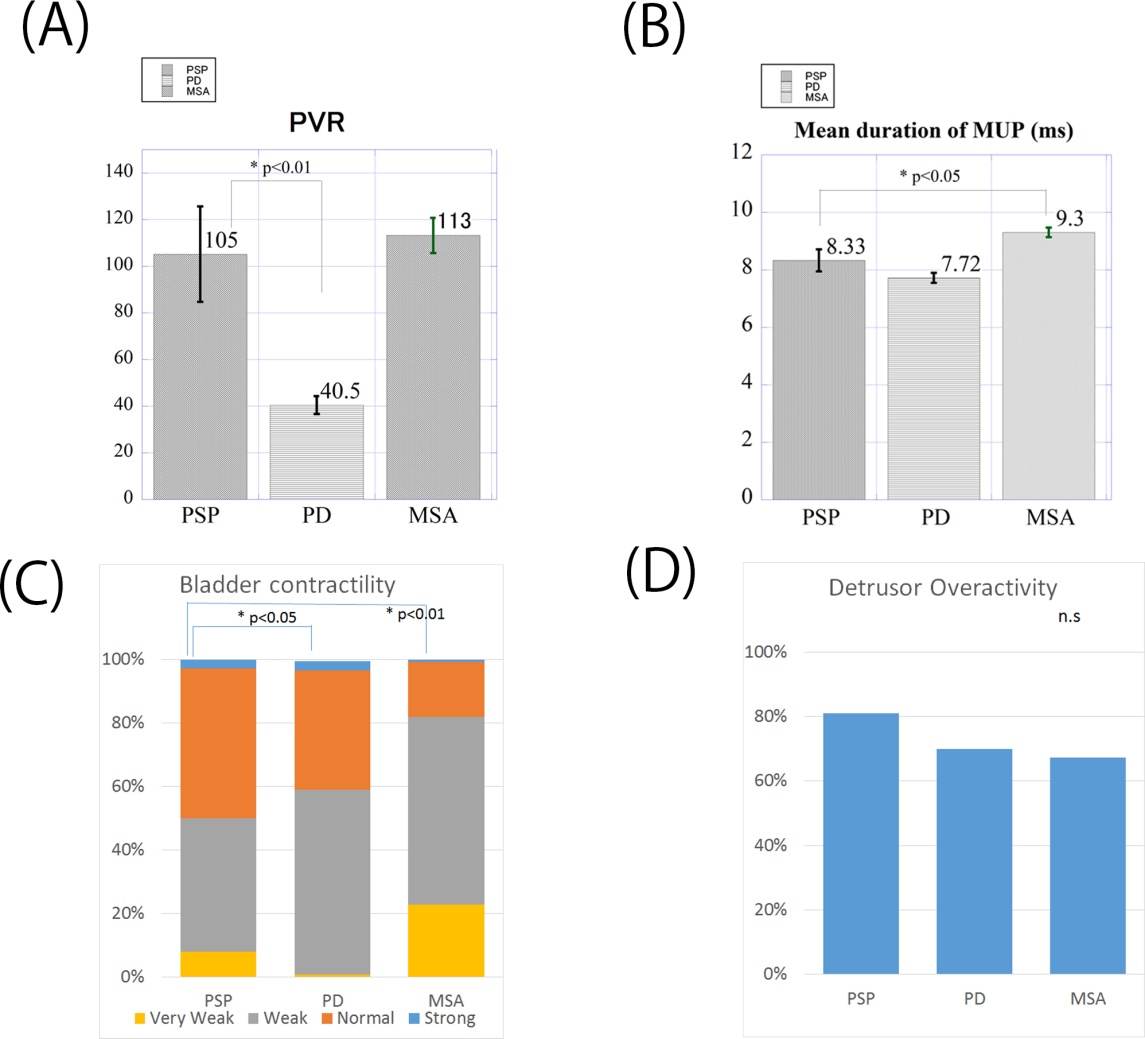
Urodynamic study and EAS-EMG of patients with PSP, PD, and MSA. The postvoid residual volume (**A**), mean duration of MUP by EAS-EMG (**B**), bladder contractility evaluated by Schafer’s nomogram (**C**), and the prevalence of detrusor overactivity in patients with PSP, PD, and MSA are depicted. Abbreviations: MUP, motor unit potential; EAS-EMG, external anal sphincter electromyography; MSA, multiple system atrophy; PD, Parkinson’s disease; PSP, progressive supranuclear palsy.

Urodynamic study showed that 81% of patients with PSP exhibited DO, which was slightly higher than that detected in patients with PD (69%) and MSA (67%); however, there was no statistically significant difference among the three patient groups (Fig 2D). The prevalence of DSD was 13.9% in patients with PSP, which was slightly higher than that in patients with PD (6.4%) and slightly lower than that in patients with MSA (19.1%); again, there was no statistical significance among the three patient groups. The PVR of patients with PSP was 105 ± 18 ml, which was significantly larger than that of patients with PD (40 ± 3.8 ml) (*P < 0.01, Fig 2A) and was almost equivalent to that of patients with MSA (113 ± 7.5 ml). Pressure flow study revealed that the bladder contractility of patients with PSP was not statistically different from that of patients with PD and was significantly better than that of patients with MSA (*P < 0.05, Fig 2C). The mean duration of MUPs in patients with PSP was 8.3 ± 0.3 ms, which was not statistically different from that in patients with PD (7.7 ± 0.17 ms) and was significantly shorter than that in patients with MSA (9.3 ± 0.16 ms) (*P < 0.05, Fig 2B).

### Relationship among the FAB scores, MMSE, disease duration, Hoehn and Yahr scale, and urinary dysfunction in patients with PSP

The FAB scores showed a significant inverse correlation with the mean duration of MUP as determined by EAS-EMG (*r* = −0.408, *P* = 0.025), whereas the PVR did not significantly correlate with the FAB scores (Table 1). MMSE, mean disease duration, and Hoehn and Yahr scale did not show significant correlations with the mean duration of MUP or PVR (Table 1). Furthermore, the FAB scores, MMSE, mean disease duration, and Hoehn and Yahr scale did not correlate with bladder contractility as evaluated by Schäfer’s nomogram (Table 2). The mean disease duration in patients with PSP without DO was significantly shorter than in those with DO (Table 3). The FAB scores, MMSE, and Hoehn and Yahr scale were not significantly related to the presence or absence of DO (Table 3).

**Table 1:** Relationship between the FAB score and urinary dysfunction. Correlation among the FAB score, MMSE, mean disease duration, Hoehn and Yahr scale, PVR, and mean duration of MUPs.

		FAB	MMSE	Mean duration	Hoehn and Yahr
PVR	r	−0.029	0.025	0.026	0.284
	*P*	0.880	0.891	0.879	0.080
Mean duration of MUPs	*r*	−0.408*	0.123	0.033	0.030
	*P*	0.025	0.526	0.853	0.863

*P < 0.05

Abbreviations: FAB, frontal assessment battery; MMSE, mini-mental state examination; MUP, motor unit potential; PVR, postvoid residual.

**Table 2:** Relationship among the FAB score, MMSE mean disease duration, Hoehn and Yahr scale, and the degree of bladder contractility. Data are expressed as means ± standard error of mean.

Bladder contractility	FAB	MMSE	Mean duration	Hoehn and Yahr
Very weak (*n* = 3)	12.33 ± 0.66	23.33 ± 2.60	2.00 ± 0.57	2.66 ± 0.33
Weak (*n* = 13)	10.31 ± 1.1	23.33 ± 1.22	3.63 ± 0.51	3.18 ± 0.24
Normal (*n* = 14)	10.21 ± 0.86	22.40 ± 1.40	2.52 ± 0.37	3.11 ± 0.25

FAB score, MMSE, mean disease duration, and Hoehn and Yahr scale did not significantly differ in relation to the degree of bladder contractility.

**Table 3:** Correlation among the FAB score, MMSE, mean disease duration, Hoehn and Yahr scale, and the presence or the absence of DO. Data are expressed as means ± standard error of mean.

DO	FAB	MMSE	Mean duration*	Hoehn and Yahr
+ (*n* = 24)	10.88 ± 0.68	22.29 ± 1.01	3.23 ± 0.33	3.14 ± 0.17
− (*n* = 8)	11.13 ± 1.1	24.57 ± 1.25	1.71 ± 0.35	3.12 ± 0.29
*P* value	0.85	0.28	0.006*	0.95

Abbreviations: DO, detrusor overactivity

* p<0.05

## Discussion

The present study demonstrated that urinary storage symptoms in patients with PSP were not statistically different from those observed in patients with PD and MSA, whereas urinary voiding symptoms in patients with PSP were significantly milder than those experienced by patients with MSA, but were almost equivalent to those detected in patients with PD. The prevalence of DO in patients with PSP was slightly higher than that of patients with PD and MSA, without being statistically significant. Although the PVR of patients with PSP was significantly higher than that of patients with PD and almost equivalent to that of patients with MSA, the bladder contractility of patients with PSP as evaluated by Schäfer’s nomogram was equivalent to that of patients with PD and was significantly better than that of patients with MSA. There were discrepancies in the results of PVR and Schäfer’s nomogram in this study. We found that the mean disease duration in patients with PSP without DO was significantly shorter than those with DO, and the FAB scores showed a significant inverse correlation with the mean duration of MUPs as determined by EAS-EMG.

The present results suggest that most patients with PSP might have both urinary storage and voiding dysfunctions; however, the exact prevalence and severity of urinary dysfunction in patients with PSP remain uncertain. In general, autonomic dysfunction is not a characteristic feature of PSP, and early unexplained dysautonomia that is characterized by marked hypotension and urinary disturbances is a mandatory exclusion criterion according to the NINDS-SPSP criteria [8]. This may precipitate a misunderstanding that the diagnosis of PSP is unlikely if patients with Parkinsonism also demonstrate urinary symptoms. For example, the co-occurrence of urinary incontinence and poor L-DOPA-responsive Parkinsonism fulfill the criteria for diagnosis of probable MSA and do not exclude the diagnosis of PSP-P. Patients with PSP-P are frequently misdiagnosed as PD due to the L-DOPA responsiveness, at least in the early stages [1, 2]. In addition, nonmotor symptoms including urinary symptoms are observed in early-stage PD [24]. Thus, the combination of urinary dysfunction and Parkinsonism is usually misdiagnosed as PD or MSA-P.

Nevertheless, several reports have already shown that urinary dysfunction is not uncommon in PSP [4–7]. First, urinary incontinence is frequently observed in PSP [4–7], requiring urethral catheterization in late-stage disease in some patients [4]. The latency of urogenital dysfunction was associated with the disease duration, suggesting that patients with PSP with early-onset urogenital dysfunction had shorter disease duration compared with those with late-onset urogenital dysfunction [6]. Prominent urinary retention was also presented in certain PSP cases [7]. These studies provide further support for our findings demonstrating that the prevalence of urinary symptoms in patients with PSP was equivalent to that in patients with PD and MSA, in whom urinary dysfunctions are common and severe.

Importantly, several previous studies examining urinary dysfunction in PSP mainly focused on the presence or absence of urinary symptoms, especially urinary incontinence, whereas detailed urinary storage and voiding symptoms were not systematically investigated. In addition, urodynamic examination was not performed in these studies. Urodynamic study allows for the assessment of detrusor contractility and bladder outlet function. DO during the filling phase usually causes urinary urgency or urinary urgent incontinence, whereas detrusor underactivity during voiding phase might lead to urinary voiding symptoms or urinary retention. As motor and cognitive functions usually deteriorate as PSP progresses [1, 2], it is generally challenging to discern whether urinary incontinence is a result of DO or neurological dysfunction, such as gait disturbance or cognitive impairment, based on the findings from only a urinary symptoms questionnaire. Since we observed DO in a vast majority of patients with PSP in this study, most of the urinary urgency or urgent urinary incontinence might be a result of DO in our patient cohort. In addition, the present study also suggested that the prevalence of DO might be associated with the disease duration. It should be noted that some of the patients with PSP in this study had concomitant urinary voiding dysfunction, which has not been commonly observed in previous studies [4–6]. In addition, the PVR in patients with PSP was unexpectedly large in this study (105 ± 18 ml), which was significantly larger than that of PD and was almost equivalent to that of MSA. The present study revealed that patients with PSP had both urinary storage and voiding dysfunctions, and the underlying cause is still not known. These findings may be partially attributable to the frontal cortical dysfunction observed in patients with PSP [16]. Frontal cortex dysfunction such as dysexecutive syndrome is common in patients with PSP [16], which was observed in our patient cohort with PSP as detected by decreased FAB scores and changes detected by IMP-SPECT. The latter showed that 22 of 26 (84%) patients with PSP diagnosed at Chiba University who underwent this analysis showed decreased frontal cortical blood flow.

Recent functional imaging studies indicate that the frontal cortex plays a significant role in voluntary micturition control in humans [9, 10]. Although micturition is mediated by spino-bulbo-spinal reflex, voluntary control is achieved via PAG that has strong and direct connections with the frontal cortex [9, 10]. Several previous reports suggested that frontal lesions such as tumor, infarction, and vascular anomalies were associated with urinary incontinence [11]. Experimental studies also showed that electrical stimulation of the frontal cortex inhibits the micturition reflex and bladder contraction-related neurons in the prefrontal cortex of normal cats [25, 26]. Distinct subsets of neurons are excited during the bladder relaxation and contraction phase. These studies suggest that the frontal cortex, especially the prefrontal cortex, plays a significant role in regulating the micturition reflex. Although we failed to detect a significant relationship between the FAB scores and urinary dysfunction as determined by the PVR, bladder contractility, and DO, we believe that more detailed cognitive function tests and quantitative evaluation of the frontal cerebral blood flow with a larger cohort of patients with PSP are necessary to confirm the relationship between frontal and urinary dysfunction in patients with PSP. Although it is plausible that urinary storage symptoms might be caused by dysfunction of the frontal cortex which inhibit the voiding reflex during the storage phase, frontal cortical dysfunction is unlikely the sole cause of urinary voiding dysfunction. Neurodegeneration in the brain stem, a commonly observed pathology in patients with PSP [1, 2], might also be contributing to urinary voiding dysfunction in these patients. These potential mechanisms need to be further investigated in future studies.

Our findings using EAS-EMG also showed that the mean duration of MUPs in patients with PSP was significantly shorter than that in patients with MSA. Although neuronal loss in Onuf’s nucleus is well known in MSA [27], little is known about the neuropathology in this location in PSP. Scaravilli et al. reported that neuronal loss was present in Onuf’s nucleus in a clinicopathological study of three patients with a definite PSP diagnosis [28]. However, the mean duration of MUPs longer than 10 ms is considered to reflect neurogenic changes in Onuf’s nucleus [29]; thus, our EAS-EMG results do not indicate a neurogenic change in Onuf’s nucleus in patients with PSP. We found significant inverse correlations between the FAB scores and mean duration of MUPs by EAS-EMG, which suggest that those patients with longer duration of MUPs had worse frontal dysfunction and that the progression of frontal dysfunction and neurodegeneration in Onuf’s nucleus might be occurring in parallel in PSP. These points need to be examined in a larger cohort of patients with PSP in future clinical studies.

There are several limitations in this study. First, the sample size of patients with PSP was small compared with those of patients with PD and MSA and was not sufficient to assess the prevalence and severity of urinary dysfunctions in PSP. Second, we could not evaluate the correlation between gait disturbances and urinary dysfunctions. Although all patients with PSP had gait disturbances and frequent falls, we did not evaluate the severity of gait disturbances. We also evaluated motor functions only by the Hoehn and Yahr scale. Third, the assessment of frontal dysfunction in this study was not sufficient. Montreal cognitive assessment is a preferred and more sensitive method for detecting frontal dysfunction in parkinsonian disorders.

## Conclusions

The present study demonstrated that patients with PSP experienced a variety of urinary dysfunctions. Urinary storage dysfunction of PSP was not different from that of PD or MSA, whereas urinary voiding dysfunction of PSP was milder than that of MSA and more severe than that of PD. These features should be taken into account for the differentiation of PSP from PD and MSA.
